# Mutanome Engineered RNA Immunotherapy: Towards Patient-Centered Tumor Vaccination

**DOI:** 10.1155/2015/595363

**Published:** 2015-12-30

**Authors:** Mathias Vormehr, Barbara Schrörs, Sebastian Boegel, Martin Löwer, Özlem Türeci, Ugur Sahin

**Affiliations:** ^1^Research Center for Immunotherapy (FZI), Langenbeckstr. 1, Building 708, 55131 Mainz, Germany; ^2^Biopharmaceutical New Technologies (BioNTech) Corporation, An der Goldgrube 12, 55131 Mainz, Germany; ^3^TRON-Translational Oncology at the University Medical Center of Johannes Gutenberg University, Freiligrathstr. 12, 55131 Mainz, Germany

## Abstract

Advances in nucleic acid sequencing technologies have revolutionized the field of genomics, allowing the efficient targeting of mutated neoantigens for personalized cancer vaccination. Due to their absence during negative selection of T cells and their lack of expression in healthy tissue, tumor mutations are considered as optimal targets for cancer immunotherapy. Preclinical and early clinical data suggest that synthetic mRNA can serve as potent drug format allowing the cost efficient production of highly efficient vaccines in a timely manner. In this review, we describe a process, which integrates next generation sequencing based cancer mutanome mapping,* in silico* target selection and prioritization approaches, and mRNA vaccine manufacturing and delivery into a process we refer to as MERIT (mutanome engineered RNA immunotherapy).

## 1. Introduction

Somatic mutations are on the one hand a cause of cancer and drive the unlimited proliferation and malignant behavior of tumor cells. But on the other hand, the tens to hundreds of somatic nonsynonymous mutations [1] (the mutanome) displayed by a tumor are a rich source for highly specific targets for the recognition by cytotoxic and helper T cells with antitumor activity.

T cells are educated in the thymus, through a process called negative selection, to prevent the recognition of autoantigens. T cells readily recognize foreign antigens but in general are unable to recognize self-antigens, including most shared tumor antigens, with a high avidity. Mutated antigens on the other hand are not present in the thymus. Thus, the neoepitope-specific T cell repertoire is not affected by negative selection. Furthermore, as mutated antigens are only expressed in cancer cells, neoantigen-specific T cells would not cause on-target effects on healthy tissue. This renders mutated antigens ideal targets for therapeutic vaccination.

The importance of neoantigens in the rejection of transplantable murine tumors had already been recognized in the 1970s by Boon and colleagues [2, 3]. Only recently, however, the concurrence of technological and scientific breakthroughs has opened the way for exploitation of mutations for the development of truly personalized, mutation specific T cell vaccines. While deciphering the first human genome took about 13 years with a cost of about $2.7 billion [4], advances in next generation sequencing (NGS) make it possible today to sequence a genome, exome, or transcriptome within hours for approximately $1,000 [5]. This paved the way for a deeper understanding of neoantigen-specific T cells in cancer. Consequently, in 2012, we suggested that the mutanome could be exploited for tumor vaccination [6, 7]. Our team provided the preclinical proof of concept that NGS based mutation identification, followed by bioinformatic target selection and prioritization, could be utilized to produce a therapeutic vaccine that is effective in mice [6]. By now, several other groups demonstrated therapeutic efficacy of personalized vaccines with similar approaches [8–11]. Yadav and colleagues used mass spectrometry to select potential neoepitopes expressed on MHC class I molecules [9]. As pointed out by the authors, the complexity of mass spectrometry hampers its utility in a clinical setting.

Recent studies have further indicated the importance of neoantigen-specific T cells in the response against human tumors. Brown and coworkers showed that predicted neoepitopes, as well as CD8 and HLA-A expression, correlates with increased survival across different cancer types [12]. Furthermore, Snyder et al. [13, 14] and Tran et al. [15] recently demonstrated that mutation specific T cells play a pivotal role in the therapeutic efficacy of immune checkpoint blockade.

## 2. Concept

**Figure 1:**
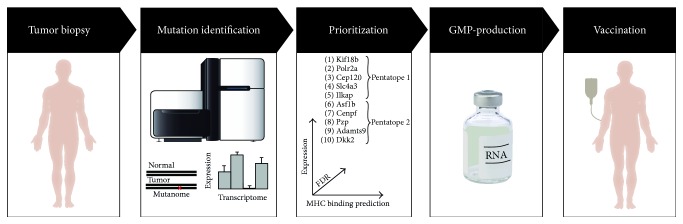
Concept of mutanome engineered RNA immunotherapy (MERIT). Next generation sequencing of nucleic acid from a tumor biopsy and healthy tissue is used to identify expressed, nonsynonymous, somatic mutations. Vaccine targets are selected based on several parameters such as expression, their MHC binding prediction, and restriction as well as a false discovery rate (FDR) [16]. Mutations encoded on pentatope RNAs are produced under GMP conditions and used for therapeutic vaccination.

Putting the concept of personalized cancer vaccination into practice involves a step-wise process (Figure 1).

The tumor biopsy as source for the individual patient's DNA and RNA is retrieved. By comparison of exome sequencing data of healthy tissue and tumor DNA somatic nonsynonymous mutations are identified. Transcriptome sequencing of tumor RNA then provides information on the expression levels of identified mutations. Those neoantigens which are likely to induce a T cell response are to be selected. A vaccine encoding the targets of interest is manufactured, which finally is delivered to professional antigen-presenting cells such as dendritic cells (DCs) in combination with an adequate adjuvant. Each of these steps is critical for obtaining efficient and sustained immune responses and will be discussed in more detail in Sections 2.1 to 2.4.

### 2.1. Mutation Identification

NGS analysis of DNA and RNA for mutation detection requires a representative tumor and a healthy tissue sample. A blood sample is an easy to obtain source for healthy tissue and a few mL are sufficient for NGS analysis. Patients' tumors are rarely banked as fresh frozen samples. Therefore, we set up an optimized protocol for the efficient and reproducible isolation and NGS analysis of small amounts of nucleic acid from formaldehyde-fixed, paraffin-embedded (FFPE) samples as used in routine pathology. A few sections from FFPE blocks are sufficient and in our hands additional biopsies beyond those taken as part of routine diagnostic are not required. Generally speaking, mutation evaluation is error-prone and data depends on the algorithms used [16–18]. Moreover, correct identification of mutations within tumor samples may be compromised, by, for example, tumor heterogeneity and contaminations with healthy tissue or necrotic cells. For this reason we established a statistical value to gauge the false discovery rate (FDR) and accurately discriminate true mutations from erroneous calls [16].

### 2.2. Target Selection

Tumors of patients display hundreds of mutations. Selecting the right ones as neoantigens for vaccination is challenging and critical, as not every mutation is immunogenic. We perform* in vitro* immunogenicity testing of candidate mutations with blood samples of patients. These bioassays identify prevalent immune responses of the patient and thereby validate immunogenicity of mutations of interest. Against many mutations, however, there are no spontaneous immune responses in the patient and their potential as vaccination target is not easily assessable [19]. We address this by developing tools for* in silico* selection of targets. Research in murine tumor models revealed that, surprisingly, most immunogenic mutations are MHC class II restricted. Those CD4^+^ T cells of a T_H_1 subtype are attractive effectors as they were shown to exert a potent antitumoral effect [15, 19, 20]. By applying thresholds for MHC class II binding prediction and mRNA expression levels, we were able to enrich immunogenic MHC class II-restricted epitopes. Such purely* in silico* predicted mutations used for vaccination without further validation by immunogenicity testing result in efficient and sustained control of advanced tumors in mouse models. Vaccines based on mutations selected for abundant expression only did not succeed in controlling those tumors [19].

So far, the majority of groups focused on the selection of mutations for immunization based on their property to be recognized by CD8^+^ T cells, which appear to be rare [8, 21–23] as compared to CD4^+^ T cell recognized ones. In comparison to cytotoxic CD8^+^ T cells that directly kill tumor cells, CD4^+^ T cells have a “catalytic” function within the immune system. CD4^+^ T cells orchestrate the activity of many other cell types including macrophages, NK cells, B cells, DCs, and CD8^+^ T cells or act on tumor cell survival by intratumoral secretion of inflammatory cytokines like IFN-*γ* [19, 24–26]. This further encourages approaches including mutations capable of inducing CD4^+^ T cell responses into a mutation-based vaccine on top of MHC I binding ones.

### 2.3. Vaccine Format and Production

**Figure 2:**
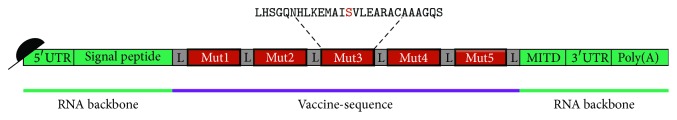
Structure of the pentatope RNA vaccine. Several modifications in the 5′ cap, 5′ and 3′ untranslated regions (UTR), poly(A) tail, and codon usage increased the translation efficiency and stability of the mRNA [30, 31]. Mutated sequences (Mut1–5) encoding 27 amino acids with the mutation in the center (red letter) are separated by nonimmunogenic 10 mer linkers. The antigen encoding sequences are flanked by a signal peptide and the MHC class I trafficking domain (MITD, transmembrane, and cytoplasmic domain of MHC class I) to ensure optimal antigen presentation [32].

Effective personalized tumor vaccination requires a suitable vaccine format that bundles the following features: safety, cost-efficiency, and scalability under GMP conditions, stability, and, most importantly, a reliable induction of a proper T cell response that depends on the adjuvant capacity and targetability into antigen-presenting cells. In this regard, synthetic* in vitro* transcribed (IVT) mRNA is appealing for several reasons. A number of clinical studies proved the safety of mRNA vaccines per se [27, 28]. Furthermore, high amounts of IVT RNA can be manufactured at reasonable cost under GMP conditions, including synthesis of the DNA template,* in vitro* transcription, purification, and quality control. For clinical grade RNA as an investigational medicinal product, the correct identity, integrity, sterility, quantity, and functionality need to be monitored. mRNA can be stable for years in the absence of RNases. The rather short half-life of IVT mRNA, due to degradation by RNases, has so far compromised the direct* in vivo* use of RNA for vaccination. For this reason, mRNA vaccines are usually delivered via adoptive transfer of* in vitro* electroporated DCs [29]. Under GMP conditions and in large scale, this process is demanding and costly. In order to allow for direct use* in vivo*, our group introduced several improvements (e.g., 5′ cap modifications, stabilizing UTR sequences, and modified poly(A) tails). This leads to increased stability and translational efficacy of synthetic IVT mRNA by several thousandfold as compared to conventional mRNA resulting in expression of the respective antigen for days [30, 31]. Moreover, we introduced routing signals which enrich the encoded antigens in MHC I as well as MHC II loading compartment and thereby result in superior CD8^+^ and CD4^+^ T cell responses [32]. A further advantage of IVT mRNA is that several mutated epitopes can be easily encoded on the same RNA molecule. Multi-epitope formats could be beneficial for priming of CD8^+^ T cells through improved CD4^+^ T cell-mediated DC licensing [26]. Moreover, a polyclonal effector T cell response may act synergistically and allows addressing tumor heterogeneity and immune escape mechanisms. We therefore use synthetic RNA encoding five neoepitopes comprising selected MHC class I as well as MHC class II binders separated by nonimmunogenic 10 mer glycine/serine linkers (Figure 2). We demonstrated that such a “pentatope” RNA is more immunogenic than the mixture of equal amounts of the respective single epitopes encoded by RNA [19]. Synthetic mRNA as a class of drug was reviewed in more detail by Sahin and coworkers [33].

### 2.4. Vaccine Delivery and T Cell Priming

T cells are primed by “professional” antigen-presenting cells such as DCs in secondary lymphoid tissue like the spleen and lymph nodes. T cell priming requires the presentation of processed peptides of the antigen by MHC class I or class II molecules of a DC to a T cell receptor on T cells. Only in combination with a costimulatory signal by immunogenic DCs are T cells activated. In addition to the costimulatory signal, the cytokine milieu during the priming phase determines the fate of T cell differentiation [34]. Thus, delivery of the vaccine into sites of T cell priming and codelivery of an adjuvant that activates DCs to upregulate costimulatory molecules and secrete the appropriate cytokines are key for obtaining a meaningful vaccine response. IVT mRNA not only encodes the antigen but acts as a DC-maturating adjuvant as well. mRNA triggers inflammation by activation of several pattern recognition receptors such as Toll-like receptors (TLR) 3, 7, and 8, retinoic acid-inducible gene 1 (RIG-I), protein kinase R (PKR), and melanoma differentiation-associated protein 5 (MDA5) [33]. This results in the maturation of immunogenic DCs and secretion of the proper cytokines for priming of antitumor T_H_1 cells and cytotoxic CD8^+^ T cells.

In order to deliver the mRNA vaccine into DCs* in vivo*, we established two different methods. One is the direct injection of naked mRNA into the lymph node, where the RNA is taken up selectively and efficiently by DCs via macropinocytosis [35, 36]. We showed in mice that in terms of expansion of antigen-specific T cells, this approach was superior to subcutaneous, intradermal, and near nodal vaccinations [35]. The other is intravenous administration of a nanoparticle formulation [19]. This formulation encapsulates the synthetic RNA, thereby protecting it from degradation and thus optimizing its bioavailability. In addition, the IV route ensures systemic delivery specifically in antigen-presenting cells, most importantly spleen-resident antigen-presenting cells. Alternatively, RNA can be administered intradermally [37] or intramuscularly [38]. All these administration routes are currently being tested in various preclinical and clinical studies (NCT01684241, NCT02410733).

Upon selective uptake by DCs into the cytosol [36], translation of the mRNA starts immediately [33]. Cytosolic proteins are usually C-terminally processed via the proteasome and transported by the TAP transporter into the endoplasmic reticulum (ER), where peptides can be further N-terminally truncated and loaded onto MHC class I molecules. CD4^+^ T cell epitopes commonly derive from extracellular proteins loaded onto MHC class II molecules in the late endosome [39]. To ensure optimal antigen presentation of encoded proteins, not only for CD8^+^ but also for CD4^+^ T cell epitopes, we flanked the target sequences with a signal peptide and the trafficking domain (transmembrane and cytosolic domain) of MHC class I. The fusion protein is routed into the ER membrane from which it travels via the Golgi apparatus to the cell membrane and back, until it is degraded and loaded onto MHC class I or MHC class II molecules. This leads to increased antigen presentation, resulting in enhanced CD4^+^ and CD8^+^ T cell responses [32].

## 3. Preclinical and Clinical Proof of Concept

The first preclinical proof of concept for the mutanome engineered RNA immunotherapy (MERIT) integrating the above described aspects into one process was obtained in 2012 [6]. Sequencing of DNA, as well as RNA, of the C57BL/6-derived B16F10 melanoma cell line in comparison to healthy tissue revealed hundreds of targetable mutations. Immunogenicity and mouse tumor treatment studies with the mutations presented as peptide as well as mRNA vaccine format revealed that more than a third of the identified mutations were recognized by T cells (16/50) and that a fraction of these T cell responses were associated with tumor growth control and survival benefit in immunized mice. In CT26 and 4T1 tumor models of BALB/c background we confirmed that mutations are frequently immunogenic (21–45%) and capable of inducing meaningful control of advanced tumors in mice. Recently, MERIT entered trials in cancer patients and the lessons learned in the preclinical models were translated into the human setting. In the meantime, clinical trials exploring adoptive T cell transfer [15, 21, 40] or checkpoint blockade [11, 13, 14, 23, 40] data are supporting the notion that mutation recognizing T cells play a pivotal role in the therapeutic effect. Moreover, concepts similar to the MERIT approach, exploiting peptide or DC vaccines, proved successful as well [8, 9, 11, 22]. In a first-in-human clinical study, in which according to the MERIT approach an actively individualized mutation-based vaccine is manufactured for each and every patient (“IVAC mutanome,” Phase I, NCT02035956) and administered intranodally, we are currently assessing the safety and tolerability, as well as the induction of cellular immune responses in melanoma patients. To bridge the time required for manufacturing of their individual mutanome vaccine, patients with positive tumors start with an mRNA vaccine encoding two shared antigens (NY-ESO-1 and Tyrosinase) and continue with the vaccine targeting ten of their mutations encoded on two penta-epitope (pentatope) RNAs [19]. As the assessment of treatment-emergent T cell responses is end-point relevant, scientifically sound and qualified bioassays (e.g., ELISpot, flow cytometric cytokine release, and tetramer technology) have to be used according to good clinical laboratory practice (GCLP) standards and documented according to MIATA [41].

## 4. Conclusions

Cancer therapy is moving from a drug-centered to a patient-centered paradigm. The MERIT approach integrates several highly innovative technologies into a process for a universally applicable, but truly personalized, tumor treatment and therefore initiates a paradigm shift in cancer therapy. Research in murine tumor models raises hope that this concept will be effective in humans as well and first clinical results seem promising. We believe that neoantigen-targeting immunotherapies, probably in combination with other therapies such as checkpoint blockade, will become a relevant part of cancer treatment in the near future.
